# It is not always child abuse: multiple fractures due to hypophosphatemic rickets associated with elemental formula use

**DOI:** 10.1002/ccr3.1052

**Published:** 2017-07-07

**Authors:** Kamal Abulebda, Samer Abu‐Sultaneh, Riad Lutfi

**Affiliations:** ^1^ Department of Pediatrics Section of Pediatric Critical Care Medicine at Indiana University School of Medicine Riley Hospital for Children at Indiana University Health Indianapolis Indiana

**Keywords:** Child abuse, elemental formula, hypophosphatemic rickets, rickets

## Abstract

Rickets is not a disease of the past. We described a toddler who developed hypophosphatemic rickets associated with the use of elemental formula. This case highlights the importance of frequent monitoring of mineral metabolism in children receiving elemental formula and considering rickets in the workup of child abuse.

## Case Presentation

A two‐year‐old girl with a complicated past medical history significant for Pierre Robin sequence, hypoxic ischemic injury at birth, global developmental delay, recurrent pneumonia, and feeding difficulties required elemental formula (EF). Due to a history of maternal drug abuse during pregnancy, the patient's mother lost custody and the patient was placed in foster care with her great‐aunt. The patient had bilateral femur, right humeral, and left proximal tibia fractures at the age of 12, 16, and 20 months, respectively, which raised the suspicion of child abuse. Investigations by Child Protective Services did not reveal any clear evidence of serious or immediate risk of harm to the child by great‐aunt. However, given the open investigation in this case, the great‐aunt was not able to proceed with the adoption process of the child.

**Figure 1:**
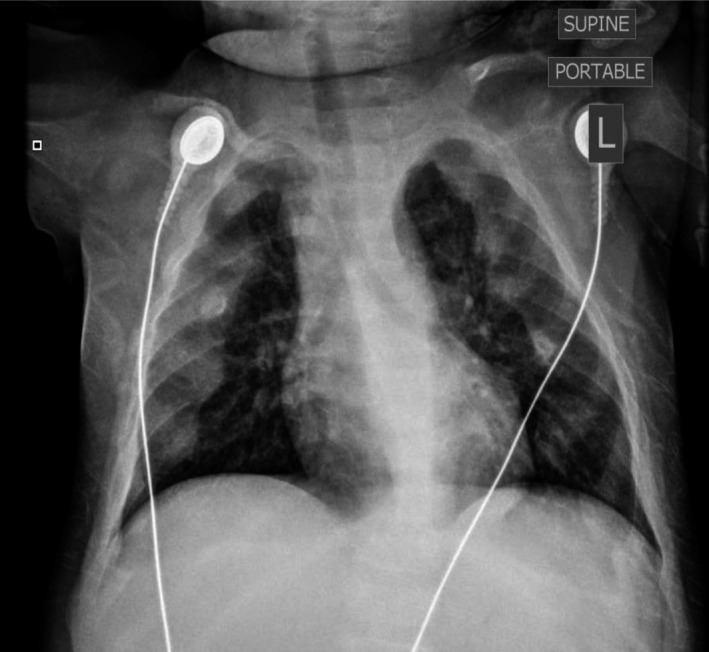
Chest radiograph at admission shows enlarged and confluent appearance of the anterior ribs at the costochondral joints bilaterally consistent with rachitic rosary.

**Figure 2:**
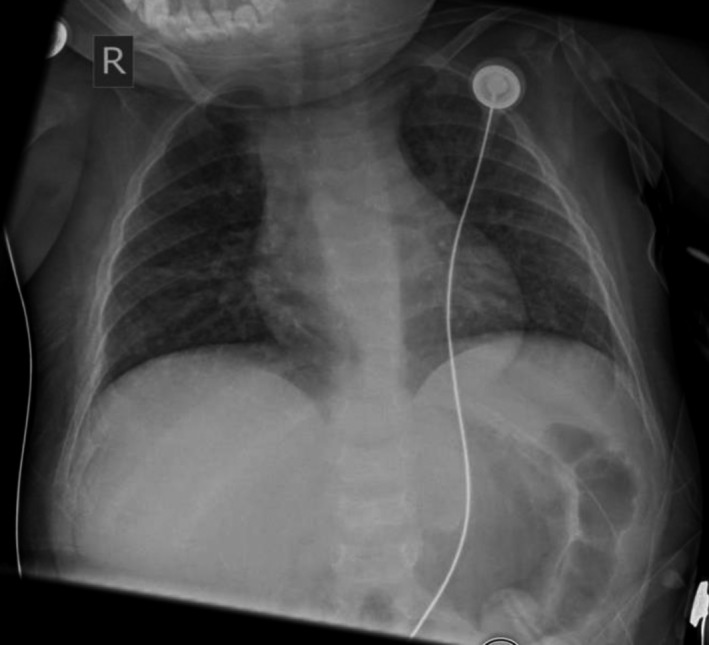
Chest radiograph 5 months prior to this hospitalization with chronic interstitial pulmonary changes.

**Figure 3:**
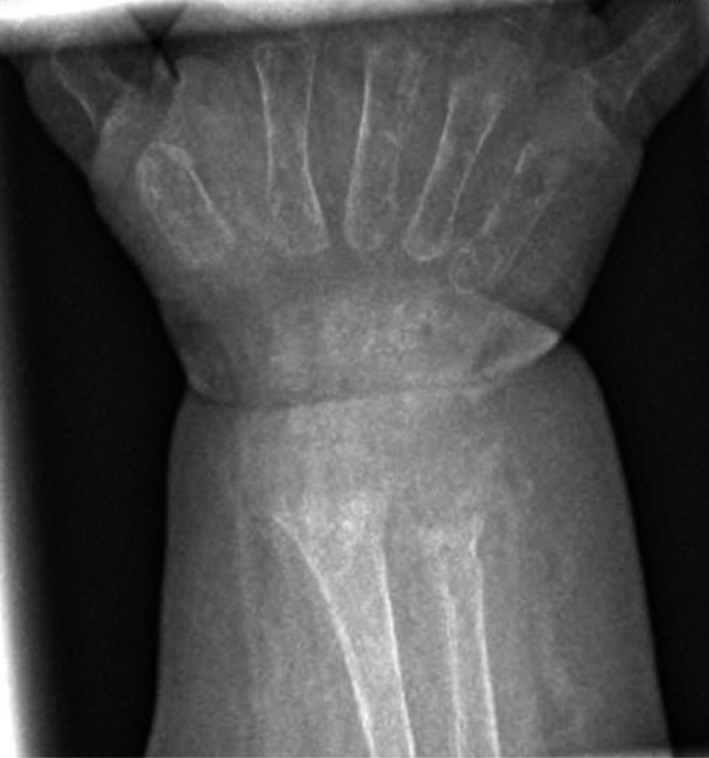
Radiographic image at admission of the wrist shows generalized osteopenia with cupping and fraying of metaphysis consistent with rachitic changes.

The patient was admitted to our pediatric intensive care unit with respiratory distress requiring placement on high‐flow nasal cannula. The chest radiographic on admission showed enlarged and confluent appearance of the anterior ribs at the costochondral joints bilaterally consistent with rachitic rosary (Fig. 1). This finding was different from her previous images 5 months prior to this hospitalization (Fig. 2). Further investigations, after stabilization, included wrist imaging which were consistent with rickets and generalized osteopenia with cupping and fraying of metaphysis (Fig. 3). Laboratory work was consistent with hypophosphatemic rickets likely due to EF (neocate) usage. The patient responded rapidly to phosphate supplementation both clinically and radiographically.

## Discussion

Fractures are a common presentation in child abuse and are observed in 25–50% of abused children 1. However, fractures are also the most common type of accidental childhood injuries accounting for 8–12% of pediatric injuries 2. The suspicion of child abuse, when the injuries are limited to the skeletal system, should lead to attempts to find an alternative cause of injury, particularly osteogenesis imperfecta and rickets 3. In nonambulatory infants in particular, certain fracture types have a strong association with abuse. Rib fractures are strongly suggestive of abuse, especially if they are multiple with different stages of healing. Healing rickets can look like healing fractures which commonly leads to a misdiagnosis of child abuse 4.

Rickets is a disease that occurs during childhood and results from a mineralization failure of the growing bone. Many skeletal and radiographic changes can occur because of the lack of calcified osteoid and the buildup of unossified cartilage 5. Proper bone formation requires a complex interplay of several organs and chemicals. Vitamin D plays a major role as a disturbance in its production, absorption, or metabolism which is paramount in the development of rickets 6.

Other causes of rickets are inadequate sunlight exposure or as a result of a nutritional deficient such as inadequate intake of calcium and phosphorus. Vitamin D deficiency rickets is common in developed countries in infants who are solely breastfed, patients who are dark skinned, or have limited sunlight exposure 7, but rickets due to vitamin deficiency is uncommon. On rare occasions, nutritional rickets can be due to hypophosphatemia secondary to use of hydrolyzed infant formula 8. Nutritional rickets is usually treated by replacing the deficient nutrient. Breastfeeding mothers should be encouraged to start their infants on vitamin D supplements as early as the first 2 months of life to prevent nutritional rickets. Other types of rickets are vitamin D‐dependent or vitamin D‐resistant. Vitamin D‐dependent rickets, type I, results from abnormalities in the gene coding for 25(OH)D3‐1‐a‐hydroxylase, while type II results from defective vitamin D receptors. Vitamin D‐resistant rickets are either X‐Linked hypophosphatemic (familial hypophosphatemic) rickets or hereditary hypophosphatemic rickets with hypercalciuria 9 (Table 1).

**Table 1:** Types of rickets; clinical features, laboratory findings; and treatments

Type	Cause	Laboratory findings	Treatments
Nutritional rickets			
Vitamin D deficiency	Inadequate sunlight exposure, solely breastfed, or dark skinned	Serum studies: Calcium, phosphorus, alkaline phosphatase, intact parathyroid hormone, urea nitrogen, creatinine, and calcidiol	Replacing the deficient nutrients
Calcium deficiency	Diet deficient in calcium, malabsorption syndromes	Urine studies: Urinalysis and levels of urinary calcium and phosphorus	
Phosphorus deficiency	Diet deficient in phosphorus, malabsorption syndromes		
Vitamin D‐dependent rickets			
Type I	Renal 25(OH)D3‐1‐a‐hydroxylase deficiency	Same evaluation as nutritional rickets	Calcitriol
Type II	Defective interaction between 1,25‐dihydroxyvitamin D3 (calcitriol) and receptor	Elevated levels of circulating calcitriol differentiate this type from type I	Calcitriol and calcium
Vitamin D‐resistant rickets			
X‐linked hypophosphatemic rickets (familial)	Impaired proximal renal tubular phosphorus reabsorption with normal calcitriol levels	Same evaluation as nutritional rickets	Oral phosphate and calcitriol
Hereditary hypophosphatemic rickets with hypercalciuria	Impaired proximal renal tubular phosphorus reabsorption with elevated calcitriol levels		Oral phosphate
Miscellaneous			
Renal rickets	Chronic renal failure	Same evaluation as nutritional rickets	Vitamin D and phosphate binding
Oncogenic rickets	Tumor‐induced inhibition of renal 25(OH)D3‐1‐a‐hydroxylase		Treat underlying malignancy
Rickets of prematurity	Multifactorial		Replacing the deficient nutrients

The skeletal changes caused by rickets are most pronounced at the knees, wrists, and anterior rib ends and can include soft ribs, an enlarged costochondral junction, and irregularly thickened growth plates of long bones. Classic radiographic findings include widening of the distal physis, fraying and widening of the metaphysis, and angular deformities of the arm and leg bones 5. The pediatric radiologist must also be familiar with the constellation of skeletal findings in both rickets and nonaccidental trauma 10.

Laboratory investigation for rickets should include serum levels of calcium, phosphorus, alkaline phosphatase (ALK), intact parathyroid hormone, and 25‐hydroxyvitamin D (calcifediol). The serum level of 25‐hydroxyvitamin D is usually indicative of the patient's overall vitamin D status 6, 9. Urine studies include urinalysis and urinary levels of calcium and phosphorus.

Our patient was placed on neocate formula due to feeding difficulties prior to this hospitalization. During this hospitalization for respiratory distress, she was found to have low‐serum phosphate level (1.1 mg/dL, normal range 3.6–6.5) with low urinary phosphate (<10 mg/dL, normal range 20–60). This reflects normal tubular reabsorption of phosphate and excluded renal losses and X‐linked hypophosphatemic rickets. Initial serum calcium, urine calcium, and serum 25‐hydroxyvitamin D were normal. Total ALK was elevated at 3777 Units/L (normal range 102–400) and bone ALK at 1399 Units/L (normal range 31–152) which indicates rapid bone turnover. The patient was placed on an oral phosphate supplement, and her formula was switched to pediasure. The patient later developed hypocalcemia which responded to an oral supplementation. After normalization of serum phosphate and calcium level, the ALK slowly corrected. Our patient had no further fractures, and bone mineralization improved radiologically. This case highlights two important teaching points: first, the importance of considering rickets in suspected child abuse cases when bone fractures are the only presentations and second is the importance to carefully monitor minerals in children fed with EF. The child's history and environment are important, especially information related to feeding and sun exposure. The collaboration among pediatric radiologists, pediatric endocrinologists, pediatric intensivists, pediatric child abuse specialists, and general pediatricians is essential for this evaluation 11.

## Authorship

KA, SAS, and RL: contributed equally to this work. RL: drafted the first draft of the article. The entire author group critically reviewed and approved the manuscript.

## Conflict of Interest

None declared.
